# Alterations of the skeletal muscle contractile apparatus in necrosis induced by myotoxic snake venom phospholipases A_2_: a mini-review

**DOI:** 10.1007/s10974-023-09662-4

**Published:** 2023-12-08

**Authors:** Alfredo Jesús López-Dávila, Bruno Lomonte, José María Gutiérrez

**Affiliations:** 1 Institute of Molecular and Cell Physiology, Hannover Medical School, Carl-Neuberg-Str. 1, 30625 Hannover, Germany; 2 Instituto Clodomiro Picado, Facultad de Microbiología, Universidad de Costa Rica, San José, 11501 Costa Rica

**Keywords:** Snake venom, Myonecrosis, Sarcomere, Hypercontraction, Skinned muscle fibers, Phospholipase A_2_

## Abstract

Skeletal muscle necrosis is a common clinical manifestation of snakebite envenoming. The predominant myotoxic components in snake venoms are catalytically-active phospholipases A_2_ (PLA_2_) and PLA_2_ homologs devoid of enzymatic activity, which have been used as models to investigate various aspects of muscle degeneration. This review addresses the changes in the contractile apparatus of skeletal muscle induced by these toxins. Myotoxic components initially disrupt the integrity of sarcolemma, generating a calcium influx that causes various degenerative events, including hypercontraction of myofilaments. There is removal of specific sarcomeric proteins, owing to the hydrolytic action of muscle calpains and proteinases from invading inflammatory cells, causing an initial redistribution followed by widespread degradation of myofibrillar material. Experiments using skinned cardiomyocytes and skeletal muscle fibers show that these myotoxins do not directly affect the contractile apparatus, implying that hypercontraction is due to cytosolic calcium increase secondary to sarcolemmal damage. Such drastic hypercontraction may contribute to muscle damage by generating mechanical stress and further sarcolemmal damage.

## Introduction

Skeletal muscle degeneration occurs in a variety of pathologies that include genetic disorders, e.g., Duchenne muscular dystrophy and other dystrophies (Saini-Chohan et al. 2012; Bargui et al. 2021), ischemia reperfusion injury (Gillani et al. 2012), cachexia (Ali and Garcia 2014), and toxic damage induced by synthetic and natural compounds (Nagahama et al. 2019; Janssen et al. 2020). Local and systemic muscle degeneration are characteristic of envenomings by a variety of snakes of the families Viperidae and Elapidae (Warrell 2010; Gutierrez et al. 2017). Local myonecrosis induced by viperid species is often associated with other tissue alterations, including vascular damage, edema, nerve damage, and blistering, and may lead to permanent tissue loss and dysfunction (Gutierrez et al. 2009). In turn, systemic myotoxicity, i.e., rhabdomyolysis, is observed in envenomings by some elapid and few viperid species. Such widespread muscle breakdown is associated with myoglobinuria, hyperkalemia, and acute kidney injury (Azevedo-Marques et al. 1985; Gutierrez et al. 2017; Johnston et al. 2022).

Snake venom-induced myotoxicity is due to the action of a variety of venom components, mainly secreted phospholipase A_2_ (PLA_2_) enzymes, PLA_2_ homologs devoid of enzymatic activity, matrix-degrading metalloproteinases, and a group of low molecular mass myotoxins having homology with β-defensins (Harris and Cullen 1990; Gutierrez and Ownby 2003; Lomonte 2023). By far, the most important myotoxic components in snake venoms are PLA_2_s and PLA_2_ homologs (Gutierrez and Ownby 2003; Lomonte and Križaj 2021). Since these toxins induce a synchronous pattern of muscle damage, they have become useful tools for studying the cellular processes involved in skeletal muscle degeneration and regeneration (Harris and Cullen 1990; Harris 2003). In particular, myotoxic PLA_2_s and PLA_2_ homologs from the venoms of the species *Bothrops asper* (family Viperidae, group II secreted PLA_2_s) and *Notechis scutatus* (family Elapidae, group I secreted PLA_2_s) have been extensively used in these investigations, although studies with other similar toxins have been also carried out (Dixon and Harris 1996; Gutierrez and Ownby 2003; Lomonte 2023). Some of these myotoxins are catalytically-active Asp-49 enzymes that cleave the *sn*-2 ester bond in phospholipids, while PLA_2_ homologs (also referred to as PLA_2_-like myotoxins) display mutations in residue 49 and other residues of the so-called calcium-binding loop, thus rendering these proteins enzymatically inactive, although keeping the ability to damage muscle fibers (Francis et al. 1991; Ward et al. 2002; Gutierrez and Ownby 2003; Lomonte and Rangel 2012).

The mechanism of action of myotoxic PLA_2_s and PLA_2_ homologs has been investigated by using a variety of experimental models (rodent in vivo tests, ex vivo muscle preparations and isolated cells, intravital microscopy, cell culture models, and phospholipid vesicles) (Dixon and Harris 1996; Gutierrez and Ownby 2003; Lomonte 2023). These investigations have shown that the first stage in the action of these toxins is the binding to the skeletal muscle plasma membrane, followed by the disruption of the integrity of muscle sarcolemma, either by enzymatic degradation of phospholipids or by a catalytically-independent destabilization of the membrane (Dixon and Harris 1996; Fernandez et al. 2013; Fernandes et al. 2014). The identity of the membrane receptors for these toxins remains largely unknown, although some candidates have been proposed (reviewed in Lomonte and Križaj (2021)).

This initial sarcolemmal destabilization leads to loss of ion gradients and depolarization (Harris et al. 2003; Melo et al. 2004), as well as to the release of muscle cytosolic enzymes, such as creatine kinase and aspartate aminotransferase (Gutierrez et al. 1984a; Preston et al. 1990). A key event in these early stages of cell damage is a prominent influx of calcium following the steep concentration gradient across the sarcolemma (Gutierrez et al. 1984a; Villalobos et al. 2007; Cintra-Francischinelli et al. 2009). The consequent increase in cytosolic calcium concentration promotes a complex series of intracellular degenerative events, such as mitochondrial degeneration, activation of calcium-dependent proteinases and cytosolic PLA_2_s, degradation of intracellular membranes, and hypercontraction of myofilaments, which bring the cell beyond the point-of-no-return and necrosis (Gutierrez et al. 1984b; Gutierrez and Ownby 2003; Harris et al. 2003; Montecucco et al. 2008). This minireview describes the main alterations of the contractile apparatus of muscle fibers as a consequence of the action of venom myotoxic PLA_2_s and PLA_2_ homologs. Further, it highlights some possible experimental strategies to be explored with the aim of reducing muscle damage, by selectively targeting key elements of the contractile mechanism.

## A prominent early alteration in myofibrillar structure is characteristic of myonecrosis induced by venom PLA_2_s and PLA_2_ homologues

Within minutes of injection of these myotoxic components in mice or rats, there is histological evidence of myofilament hypercontraction, as revealed by the formation of dense myofibrillar aggregates in the cytoplasm, leaving spaces devoid of myofibrils (Gutierrez et al. 1984b, 1991; Johnson and Ownby 1993; Harris et al. 2003). Such notorious histological change is used to estimate the fraction of fibers affected by the venom, i.e., the necrotic index, as a quantitative indicator of the myotoxic activity of venoms or toxins (Teixeira et al. 2003). At the ultrastructural level, dense amorphous masses of filaments are formed, alternating with spaces devoid of myofibrillar material. Sarcomeres are shortened and there is misalignment between Z lines (Dixon and Harris 1996; Harris et al. 2003). In other regions, the characteristic structural organization of the sarcomeres is completely lost (Gutierrez et al. 1984b; Dixon and Harris 1996). The hypercontracted masses can be also observed by scanning electron microscopy (Dixon and Harris 1996). Ultrastructural observations in muscle affected by PLA_2_-rich myotoxic venom and by purified myotoxins revealed that the plasma membrane was disrupted in areas showing hypercontraction of myofilaments (Gutierrez et al. 1984b; Dixon and Harris 1996). Histologically, these areas present ‘delta lesions’. i.e., wedge-shaped lesions associated with myofilament hypercontraction (Gutierrez et al. 1984b, 1989, 1991). This can be interpreted in the light of the early damage to the sarcolemma, generating a calcium influx that induces hypercontraction.

The dynamics of this hypercontraction process was studied by intravital microscopy by applying the venom of *Bothrops asper* and a myotoxin to the mouse cremaster muscle (Lomonte et al. 1994). Early muscle fiber contractions were followed, 3 to 4 min after application of the myotoxic agents, by the focal loss of striations and a slow retraction of myofibrils in opposite directions, generating a row of hypercontracted material alternating with empty spaces within the cells. Electron microscopic evidence shows numerous foci of hypercontracted myofibrils in a single muscle fiber (Dixon and Harris 1996).

## Does hypercontraction amplify sarcolemmal damage and necrosis?

Electron microscopic observations highlighted that the integrity of sarcolemma is interrupted in regions with hypercontracted myofibrils after injection of myotoxic PLA_2_s (Gutierrez et al. 1984b; Dixon and Harris 1996). This can be interpreted in two ways: (a) the toxins initially affect the integrity of the sarcolemma, causing a calcium influx that promotes hypercontraction, or (b) hypercontraction of myofilaments generates a mechanical stress that disrupts the sarcolemma (Dixon and Harris 1996). These possibilities are not exclusive, since an initial toxin-mediated disruption of the sarcolemma would induce a calcium influx, causing hypercontraction which, in turn, may generate mechanical stress in undamaged sarcolemma, causing its disruption, hence expanding fiber damage, a hypothesis that needs to be explored experimentally.

## Alterations in specific components of the myofibrils along the degenerative process

Immunohistochemical and immunochemical studies have addressed the changes in the immunostaining of several myofibrillar proteins along the course of the PLA_2_-induced degenerative events. There is an early loss of desmin immunostaining in necrotic fibers (Gutierrez et al. 1990; Harris et al. 2003). Desmin locates peripherally to Z discs and plays a key role in the structural and mechanical integrity of the contractile apparatus (Lazarides 1980; Paulin and Li 2004; Sweeney and Hammers 2018). Thus, desmin loss is likely to be associated with the overall disorganization of the contractile apparatus. In agreement, the degeneration of Z lines correlated with the loss of the immunostaining of desmin in muscle injected with a myotoxic venom (Vater et al. 1992). Loss of immunostaining for desmin was used to identify areas of muscle damage in venom-injected muscle (de Oliveira et al. 2023). Immunostaining of other myofibrillar proteins, i.e., α-actinin and titin, is reduced or lost at later time intervals (between 3 and 7 h), as well as dystrophin staining (Gutierrez et al. 1990; Harris et al. 2003). Likewise, staining of β-dystroglycan is affected at later time intervals, i.e., 12 h, in necrotic fibers (Vater et al. 1995). These alterations are likely to contribute to the overall disorganization of myofibrillar structure and to the loss of integration of myofibrils with the plasma membrane. In particular, the loss of α-actinin is likely to play a key role in such disorganization (Gutierrez et al. 1990).

## A shift in the ultrastructure of hypercontracted masses occurs as a consequence of the action of muscle proteinases

Histological and ultrastructural observations in skeletal muscle affected by a myotoxic PLA_2_ highlight a change in the morphology of hypercontracted myofilament masses in necrotic fibers. By 3 h and afterwards a more uniform distribution of the disorganized myofibrillar material is observed. Instead of being formed by dense hypercontracted masses alternating with spaces devoid of myofibrillar material, this material becomes more uniformly distributed in the cellular space (Gutierrez et al. 1984b, 1990). In the case of myonecrosis induced by *B. asper* myotoxic PLA_2_, such shift in myofibrillar material correlates with the loss of immunostaining of α-actinin (Gutierrez et al. 1990). α-actinin is located at the Z disc and contributes to the formation of a lattice-like structure that provides stability and integration to the contractile machinery (Sjoblom et al. 2008). Owing to the role of this protein in the mechanical integration of myofibrillar contraction, it was suggested that the redistribution of myofibrillar material is likely to be due to degradation of α-actinin, which occurs after loss of desmin. Noteworthy, when this structural shift occurs, there is no overt degradation of actin and myosin, the main components of the contractile apparatus (Gutierrez et al. 1986, 1990).

Such selective hydrolysis of key myofibrillar components, such as desmin and α-actinin, is likely due to the action of calpains, a group of cytosolic calcium-dependent cysteine proteinases that play a key role in protein turnover (Hyatt and Powers 2020). Calpains are activated by the increase in cytosolic calcium concentration in myotoxin-induced necrosis following plasma membrane disruption. Calpain-1 is known to cleave desmin in muscle atrophy (Aweida et al. 2018; Cohen 2020).

## Degradation of actin and myosin occurs at a later stage and is mostly mediated by proteinases from invading phagocytic cells

**Fig. 1:**
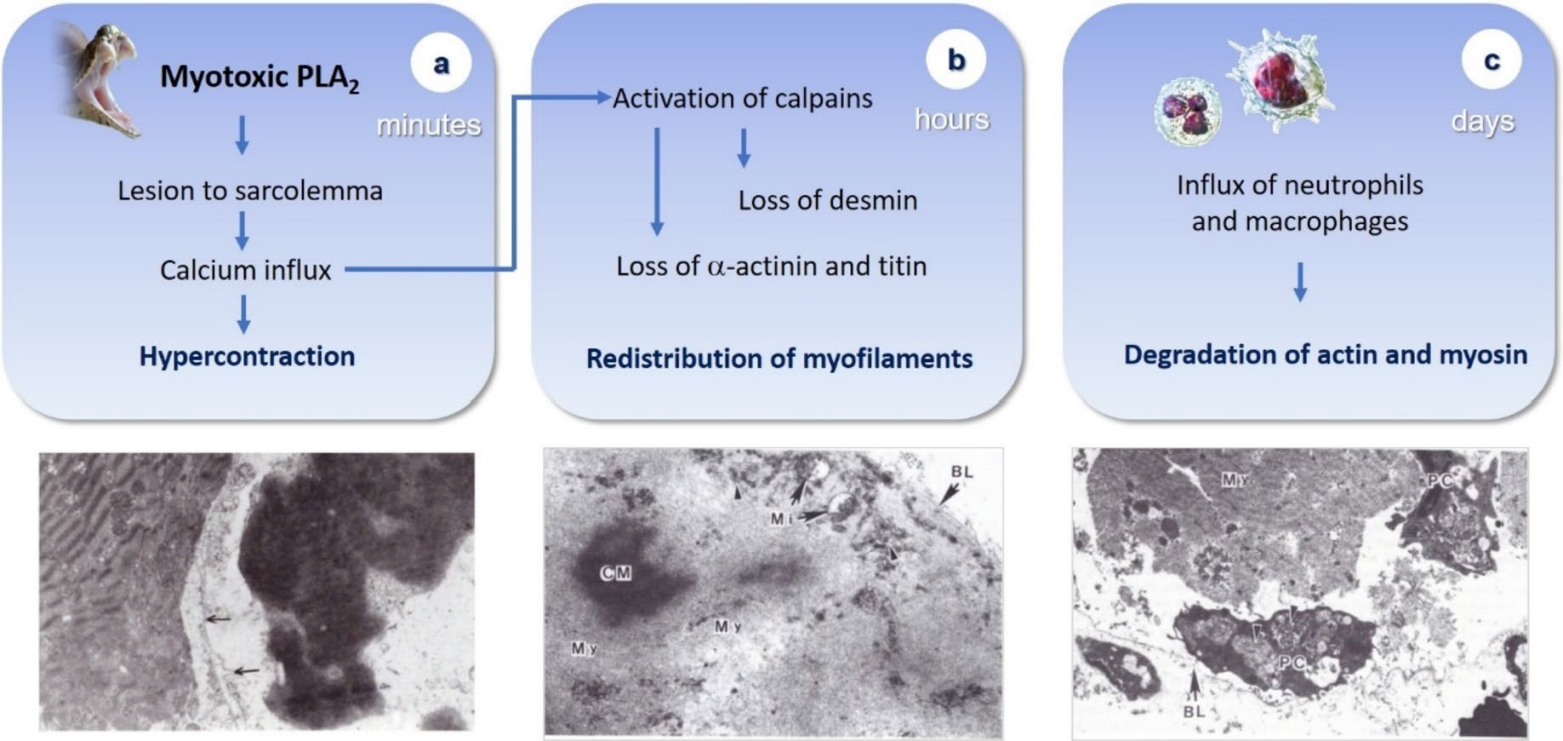
Overview of the time-course of the main alterations in myofibrillar proteins in muscle fibers affected by myotoxic PLA_2_s or PLA_2_ homologs. **a** Toxins initially bind the sarcolemma, causing membrane perturbation by catalytically dependent or -independent mechanisms. This causes a prominent calcium influx which generates hypercontraction of myofilaments and activation of calpains. **b** As a consequence, some sarcomeric proteins (desmin, α-actinin and titin, among others) are hydrolyzed. Within hours, the clumped hypercontracted myofibrillar material is redistributed in the cellular space. **c** Then, an inflammatory infiltrate (neutrophils and macrophages) invades the damaged tissue, and leukocyte lysosomal proteinases cause further degradation of sarcomeric proteins, including actin and myosin. This scheme is based on experimental studies in rodent models (see text for details). The electron micrographs shown are reprinted from Experimental and Molecular Pathology 40: 367–379 (1984) and 55: 217–229 (1991), Gutiérrez et al., with permission from Elsevier

In contrast to desmin, titin and α-actinin, the degradation of actin and myosin occurs later on in the course of the unfolding of degenerative events in skeletal muscle fibers affected by myotoxic venoms and PLA_2_s (Gutierrez et al. 1986, 1990; Abul Faiz et al. 1995). In the case of necrosis due to *B. asper* venom and myotoxic PLA_2_, SDS-PAGE analysis of muscle proteins indicates that reduction in the intensity of bands having the molecular masses of actin and myosin occurred mainly after 24 h of the onset of necrosis (Gutierrez et al. 1986, 1990). This coincides with the time-course of the arrival of neutrophils and macrophages to the necrotic tissue, thus suggesting that such degradation is due to the action of lysosomal proteinases of these inflammatory cells (Gutierrez et al. 1986, 1990). This pattern of myofibrillar protein degradation fits within the ‘two step’ model proposed by Ishiura et al. (1984) for muscle degeneration induced by plasmocid, in which the early loss of α-actinin is mediated by cytosolic muscle calcium-dependent proteinases, while the degradation of actin and myosin depends on the action of proteinases from invading inflammatory cells. Figure 1 summarizes the main events occurring in the contractile apparatus of skeletal muscle fibers as a consequence of the action of myotoxic PLA_2_s and PLA_2_ homologs.

## Do myotoxic PLA_2_s and PLA_2_ homologs have a direct action on the intracellular structures of skeletal muscle?

Although a large body of experimental evidence indicates that the sarcolemma is the primary site of action of snake venom myotoxic PLA_2_s and PLA_2_ homologs, more recent observations show intracellular localization of these toxins in myotubes in culture and in muscle cells in vivo (Massimino et al. 2018; Vargas-Valerio et al. 2021). Whether this occurs by endocytosis or secondary to sarcolemmal disruption remains to be assessed. Interestingly, a PLA_2_ homolog from *B. asper* venom binds and colocalizes in myotubes with nucleolin, a nucleolar protein that is also present on the cell surface, and also pulls down this protein in extracts of membranes obtained from ex vivo skeletal muscle preparations (Massimino et al. 2018). Furthermore, this binding is relevant to the cytotoxic action of this myotoxin since nucleolin antagonists inhibit toxin internalization and cytotoxicity (Massimino et al. 2018). Thus, myotoxins can reach the intracellular space by diverse mechanisms and might affect intracellular structures and processes as part of their mechanism of action. For instance, a myotoxic PLA_2_ from *B. asper* venom inhibits Ca^2+^ ATPase and hydrolyzes phospholipids of rabbit skeletal muscle sarcoplasmic reticulum in vitro (Gutierrez et al. 1987). We have recently explored whether myotoxic PLA_2_ and PLA_2_ homolog from *B. asper* venom affect the contractile apparatus of skeletal muscle. The main results of these studies are summarized in the next section.

## Studies on skinned muscle preparations and future research

The recent finding that PLA_2_ myotoxins can be internalized into muscle cells (Massimino et al. 2018; Vargas-Valerio et al. 2021), together with the above-mentioned hypercontraction, loss of striation and loss of immunostaining for some sarcomeric proteins observed after injection of myotoxins in animal models, has raised new questions to further elucidate their mechanisms of action. Once inside the sarcoplasm, do PLA_2_ myotoxins interact with sarcomeric proteins?; do they alter the structure and mechanical properties of myofilaments?; do they directly or indirectly modify the mechanisms of force generation or relaxation? To investigate these possibilities, we followed a reductionist strategy (i.e., removing the cell membrane and studying the effect of myotoxins in skinned muscle preparations compared to intact tissue; see Batters et al. 2014; Kalakoutis et al. 2021; Lewalle et al. 2022). In parallel, we tested the effect of small molecules (i.e., myosin inhibitors; see Rauscher et al. 2018; Marston 2019) on toxin-induced hypercontraction. The combined use of these tools allows us to dissect the effects of myotoxins at key events in excitation–contraction coupling (Dirksen et al. 2022). The main results to date obtained from these efforts are briefly described below.

In a first study (Lopez-Davila et al. 2021), single rat cardiomyocytes were used as an ex vivo experimental model. Although cardiomyocytes are not the main target of these toxins in envenomings, our growing understanding of the structural, functional and pathological similarities and differences between skeletal and cardiac muscle makes cardiomyocytes a useful model to explore the mechanism of action of myotoxins (Saini-Chohan et al. 2012; Henderson et al. 2017; Sweeney and Hammers 2018; Bargui et al. 2021; Glancy and Balaban 2021). In single intact cardiomyocytes, the Lys49 PLA_2_ homolog Mt-II, from *B. asper* venom, induced disruption of the plasma membrane and increased intracellular calcium concentrations, calcium transient amplitudes, and unloaded cell shortening. Over time, these changes typically lead to irreversible hypercontraction in a dose-dependent manner (i.e., time to hypercontraction was inversely proportional to toxin concentration, on a timescale of few minutes). On the other hand, Mt-II did not alter key parameters of force development in skinned, isometrically held single cardiomyocytes, as cooperativity, rate constant and calcium sensitivity of active force development and maximal active and passive force did not differ from control measurements. Interestingly, the irreversible hypercontraction observed in intact cardiomyocytes was reversed by the myosin inhibitor para-Aminoblebbistatin (AmBleb (Varkuti et al. 2016)). This opens the possibility of using small molecular inhibitors to modulate this aspect of toxin-induced cytotoxicity (Lopez-Davila et al. 2021).

**Fig. 2:**
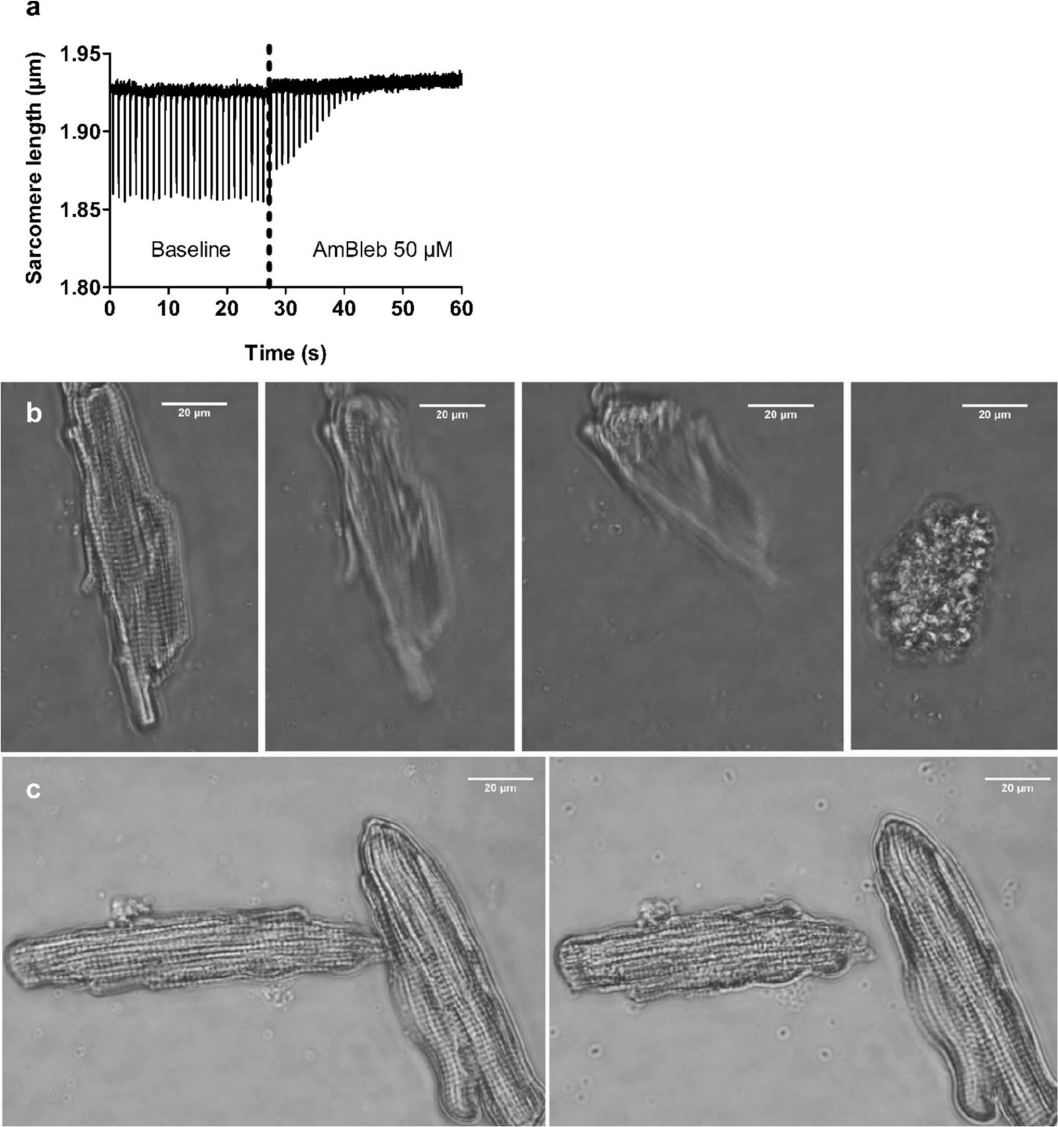
Effect of myotoxins and myosin inhibitors on unloaded, intact rat cardiomyocytes. **a** Electrically induced (1 Hz) shortening of a cardiomyocyte before and after myosin inhibition. The dotted line shows the time of administration of a bolus of HEPES solution containing the myosin inhibitor para-aminoblebbistatin (AmBleb), resulting in an intrachamber concentration of 50 μM AmBleb (the slight drift of the signal is an artifact resulting from the bolus administration). In less than 20 s, the cell stops responding to the electrical stimulus as AmBleb reaches the myosin heads, prevents myosin from entering the force-generating states, and precludes shortening. **b** Bright field microscopy of a cardiomyocyte shortly after exposure to 50 μg/mL Lys49 PLA_2_ homolog Mt-II from *B. asper* venom. Note the strong, irreversible shortening, i.e. hypercontraction. **c** Bright field microscopy of two cardiomyocytes shortly after exposure to 50 μg/mL Mt-II in the presence of 50 μM AmBleb. Despite a partial shortening (right panel in **c**), the difference from the final state in **b** is substantial. Thus, unloaded intact cardiomyocytes exposed to myotoxin were prevented from hypercontraction by the myosin inhibitor. In contrast, myotoxins did not exert any effects on skinned cardiomyocytes, nor on skinned rabbit psoas fibers (not shown). This figure was originally published in Lopez-Davila, A. J., Weber, N., Kraft, T., Matinmehr, F., Arias-Hidalgo, M., Fernandez, J., Lomonte, B., Gutierrez, J. M. (2021). Cytotoxicity of snake venom Lys49 PLA_2_-like myotoxin on rat cardiomyocytes ex vivo does not involve a direct action on the contractile apparatus. Sci Rep, 11(1), 19452. https://doi.org/10.1038/s41598-021-98594-5

In a subsequent study, we extended these observations to skeletal muscle preparations (a primary target of myotoxins), using both catalytically-active PLA_2_ (Mt-I) and inactive PLA_2_ homolog (Mt-II). Essentially, the irreversible hypercontraction observed in intact cardiomyocytes was readily reproduced after treatment with either Mt-I or Mt-II in myotubes differentiated in vitro from mouse C2C12 myoblasts. As observed for skinned cardiomyocytes, the toxins did not affect the main parameters of force development measured in single, isometrically held rabbit psoas skinned fibers (Lopez-Davila et al. 2023). The results of these studies are summarized in Fig. 2.

Studying the effects of myotoxins in skinned muscle preparations allows screening for possible effects on the full spectrum of sarcomeric proteins, thus avoiding effects that depend on sarcolemmal disruption. An additional advantage of this strategy is the access to the broadly conserved sarcomere assembly of this preparation, which is important for both the biochemistry and function of sarcomeric proteins (Lewalle et al. 2022). The lack of effect at this level implies that further elaboration of the reductionist approach, e.g., by applying myotoxins to isolated sarcomeric proteins, would not be relevant. On the other hand, the fact that the myosin inhibitor abolished hypercontraction in intact cardiomyocytes calls for its use to further explore the role of hypercontraction in the overall cell damage induced by these toxins.

As mentioned above, it has been hypothesized that myotoxins induce a high contractile force as a consequence of calcium influx, which in turn could cause mechanical damage of sarcolemma and other cellular structures, which would expand the process of cell degeneration. This raises the possibility of testing whether compounds targeting key sarcomeric proteins (e.g., myosin inhibitors or troponin modulators) would reduce the extent of muscle damage as a possible therapeutic tool. This can be addressed by using some of the methods described in this review, such as myotube cell culture, as well as ex vivo and in vivo models. Furthermore, experiments on intact, loaded muscle preparations could generate worthwhile insight. Targeting the sarcomeric proteins to correct muscle function at the level of the crossbridge cycle, as currently intended for cardiac and skeletal myopathies (Hwang and Sykes 2015), could become an emerging strategy for dealing with snake venom-induced skeletal muscle necrosis, a hypothesis that needs to be evaluated.

## Data Availability

Not applicable.
